# Accurate prostate cancer detection based on enrichment and characterization of prostate cancer specific circulating tumor cells

**DOI:** 10.1002/cam4.5649

**Published:** 2023-01-30

**Authors:** Sewanti Limaye, Simon Chowdhury, Nitesh Rohatgi, Anantbhushan Ranade, Nelofer Syed, Johann Riedemann, Darshana Patil, Dadasaheb Akolkar, Vineet Datta, Shoeb Patel, Rohit Chougule, Pradyumna Shejwalkar, Kiran Bendale, Sachin Apurwa, Stefan Schuster, Jinumary John, Ajay Srinivasan, Rajan Datar

**Affiliations:** ^1^ Sir HN Reliance Foundation Hospital and Research Centre Mumbai India; ^2^ Guy's, King's and St. Thomas' Hospital London UK; ^3^ Fortis Memorial Research Institute Gurugram India; ^4^ Avinash Cancer Clinic Pune India; ^5^ Imperial College London London UK; ^6^ Cancercare Cape Town South Africa; ^7^ Datar Cancer Genetics Nasik India; ^8^ Datar Cancer Genetics Europe GmbH Eckersdorf Germany

**Keywords:** circulating tumor cells, detection, diagnosis, immunocytochemistry, non‐invasive, prostate cancer, screening

## Abstract

**Background:**

The low specificity of serum PSA resulting in the inability to effectively differentiate prostate cancer from benign prostate conditions is a persistent clinical challenge. The low sensitivity of serum PSA results in false negatives and can miss high‐grade prostate cancers. We describe a non‐invasive test for detection of prostate cancer based on functional enrichment of prostate adenocarcinoma associated circulating tumor cells (PrAD‐CTCs) from blood samples followed by their identification by immunostaining for pan‐cytokeratins (PanCK), prostate specific membrane antigen (PSMA), alpha methyl‐acyl coenzyme‐A racemase (AMACR), epithelial cell adhesion molecule (EpCAM), and common leucocyte antigen (CD45).

**Methods:**

Analytical validation studies were performed to establish the performance characteristics of the test using VCaP prostate cancer cells spiked into healthy donor blood (HDB). The clinical performance characteristics of the test were evaluated in a case–control study with 160 known prostate cancer cases and 800 healthy males, followed by a prospective clinical study of 210 suspected cases of prostate cancer.

**Results:**

Analytical validation established analyte stability as well as acceptable performance characteristics. The test showed 100% specificity and 100% sensitivity to differentiate prostate cancer cases from healthy individuals in the case control study and 91.2% sensitivity and 100% specificity to differentiate prostate cancers from benign prostate conditions in the prospective clinical study.

**Conclusions:**

The test accurately detects PrAD‐CTCs with high sensitivity and specificity irrespective of stage, serum PSA or Gleason score, which translates into low risks of false negatives or overdiagnosis. The high accuracy of the test could offer advantages over PSA based prostate cancer detection.

## Graphical abstract

Enrichment and characterization of circulating tumor cells (CTC) can aid more effective prostate cancer detection. CTC based prostate cancer detection can reduce false negatives as well as false positives.

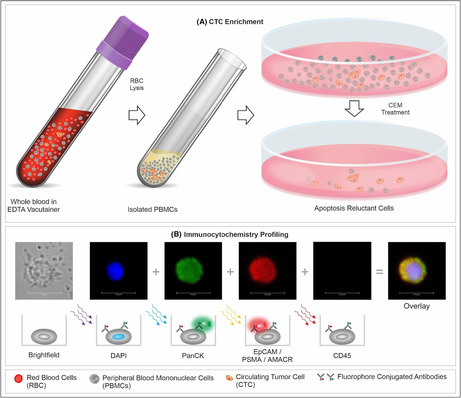

## 1 INTRODUCTION

Prostate cancer is globally the second most common malignancy and the seventh highest cause of cancer‐related mortality among men.^1^ Detection of prostate cancer at advanced stages is associated with significant morbidity and mortality as well as reduced survival, while early‐stage prostate cancer detection is associated with higher cure rate and improved survival (~99%, 5‐year^2^). At present, evaluation of serum prostate specific antigen (PSA) is part of the standard diagnostic work‐up in symptomatic cases^3^ but less suitable for prostate cancer screening in asymptomatic males due to low specificity^4^ and significant risk of false positivity^5^ which leads to overdiagnosis and overtreatment.^6^ In addition, there is a risk of false negatives, especially in advanced undifferentiated prostate cancers which may have lower PSA levels.^7^ More sensitive and specific methods which can provide for more effective prostate cancer detection are required to reduce morbidity and mortality from this disease.^8^

Circulating tumor analytes in blood have received attention for non‐radiological, non‐invasive detection of prostate cancer.^9^ Apart from serum tumor antigens, circulating tumor nucleic acids have been evaluated for prostate cancer detection but have reported limitations in sensitivity for localized prostate cancer.^10^ Circulating tumor cells (CTCs) are viable tumor derived cells in circulation, the molecular and functional evaluation of which may be comparable to that of the tumor tissue from which they originate.^11^ CTC evaluations are not prone to the limitations in sensitivity and specificity associated with circulating tumor nucleic acids or serum tumor antigens. Prior studies support the ubiquity of CTCs in prostate cancer, especially in early‐stage (localized) disease; disseminated tumor cells (DTCs) released during early stages of prostate cancer are known to remain dormant in the bone marrow and result in metastatic recurrence.^12^ In a study of bone marrow aspirates from 533 preoperative prostate cancer cases with localized disease (T2‐4, N0), DTCs were detected in 380 cases (71.3%), irrespective of pathologic stage, Gleason grade, or PSA.^13^ Another study reported CTCs in 19 (79%) of 24 treatment naïve localized prostate cancers.^14^ A third study reported >90% sensitivity in 20 known prostate cancer cases and 92.6% specificity in 27 asymptomatic men undergoing prostate cancer screening.^15^ A fourth study on pre‐operative blood from 86 prostate cancer cases reported 38.4%–62.7% CTC detection rates using CellSearch, CellCollector, and EPISPOT individually, and 80.2%^16^ when used together. In a fifth study, using a hybrid microfluidic‐imaging along with PSA immunostaining, 38–222 CTCs were reported per mL in recently diagnosed cases of localized prostate cancer.^17^ In a sixth study, using near‐infrared dyes and EpCAM immunostaining, up to 439 CTCs per mL of blood (mean: 25 CTCs/mL; median: 10 CTCs/mL) were observed in a cohort of patients with localized prostate cancer.^18^ The above studies provide evidence for the plausibility of CTC‐based prostate cancer detection. Other studies have also shown the inability of existing technology platforms to efficiently enrich and harvest sufficient CTCs. Most prior reports on CTCs in cancer are based on epitope capture using epithelial cell adhesion molecule (EpCAM) followed by immunostaining for cytokeratins (CK). A critical limitation of this approach is its acknowledged inability to effectively enrich and detect CTCs where the expression of target biomarkers such as EpCAM and CK can be significantly lower^19^, ^20^, ^21^, ^22^, ^23^ than tumor tissue or reference cell lines. Further, the expression of EpCAM and CK (as well as any other markers) may be even lower in CTCs undergoing epithelial to mesenchymal transition (EMT).^24^

We have previously described a novel functional CTC enrichment process which yields numerically sufficient CTCs for further applications.^25^ We have also shown that CTCs thus enriched from blood of patients with prostate cancer are positive for expression of PSMA, AMACR, EpCAM, and PanCK as determined by fluorescence immunocytochemistry (ICC).^26^ This multi‐marker CTC profiling has high specificity for adenocarcinomas (AD) which represent the vast majority (~92%) of prostate cancers.^27^ The test uses standardized fluorescence intensity (FI) thresholds for detection of marker positive cells, optimized to detect CTCs with a wide range of marker expression, especially those with significantly lower marker expression than tumor derived cells or PrC cell lines. In this manuscript, we report the method development as well as analytical and clinical validation of this test for prostate cancer detection.

## 2 METHODS

### 2.1 Study participants and samples

Biological samples used for method development, analytical validation, and clinical validation as described in this manuscript were obtained from participants in the following observational studies: TRUEBLOOD (http://ctri.nic.in/Clinicaltrials/pmaindet2.php?trialid=31879), ProState (http://ctri.nic.in/Clinicaltrials/pmaindet2.php?trialid=31713), and RESOLUTE (http://ctri.nic.in/Clinicaltrials/pmaindet2.php?trialid=30733). The TRUEBLOOD study (Mar 2019–ongoing) enrolls patients diagnosed with various solid organ cancers or benign (non‐malignant) conditions as well as suspected cancer cases. The ProState study (Mar 2019–ongoing) enrolls patients diagnosed with prostate cancers as well as symptomatic males suspected of prostate cancer. The RESOLUTE study (Jan 2019–ongoing) enrolls adults with neither prior diagnosis nor current symptoms suspected of cancer. All studies were approved by the Ethics Committees of the participating institutes as well as the sponsor (Datar Cancer Genetics, DCG) and are performed in accordance with the Declaration of Helsinki. Written informed consent was obtained from all study participants prior to enrolment and sample collection. Fifteen milliliters of peripheral blood were collected from all participants in EDTA vacutainers. Tumor tissue samples were obtained from TRUEBLOOD and ProState study participants who were referred for a biopsy as per Standard of Care (SoC), where such tissue sample was already available. Blood samples were also collected, after obtaining informed consent, from healthy (asymptomatic) volunteers, diagnosed cancer patients, and suspected cases who were not a part of either of the above studies but had availed of the sponsor's services. Blood samples were collected prior to the patients undergoing an invasive biopsy where the same had been advised. Blood and tissue samples were stored under refrigeration (2°C–8°C) during transport to reach the clinical laboratory within 46 h. All samples were identity masked by using blood collection vacutainers with a 10‐digit alphanumeric code. All samples were processed at the CAP and CLIA accredited facilities of the Study Sponsor, which also adhere to quality standards ISO 9001:2015, ISO 27001:2013, and ISO 15189:2012. The reporting of observational studies in this manuscript is compliant with STROBE guidelines.^28^

### 2.2 Isolation of primary tumor derived cells

The isolation of primary tumor derived cells (TDCs) from an excised tumor (malignant/benign) was performed as described previously^25^ and is also explained in Supplementary Materials.

### 2.3 Enrichment of circulating tumor cells from peripheral blood

Blood samples were processed for red blood cell (RBC) lysis and isolation of peripheral blood mononuclear cells (PBMC), following which CTCs were enriched from PBMCs as described previously.^26^, ^29^ The process is also explained in Supplementary Materials.

### 2.4 Immunocytochemistry profiling of CTCs

**FIGURE 1:**
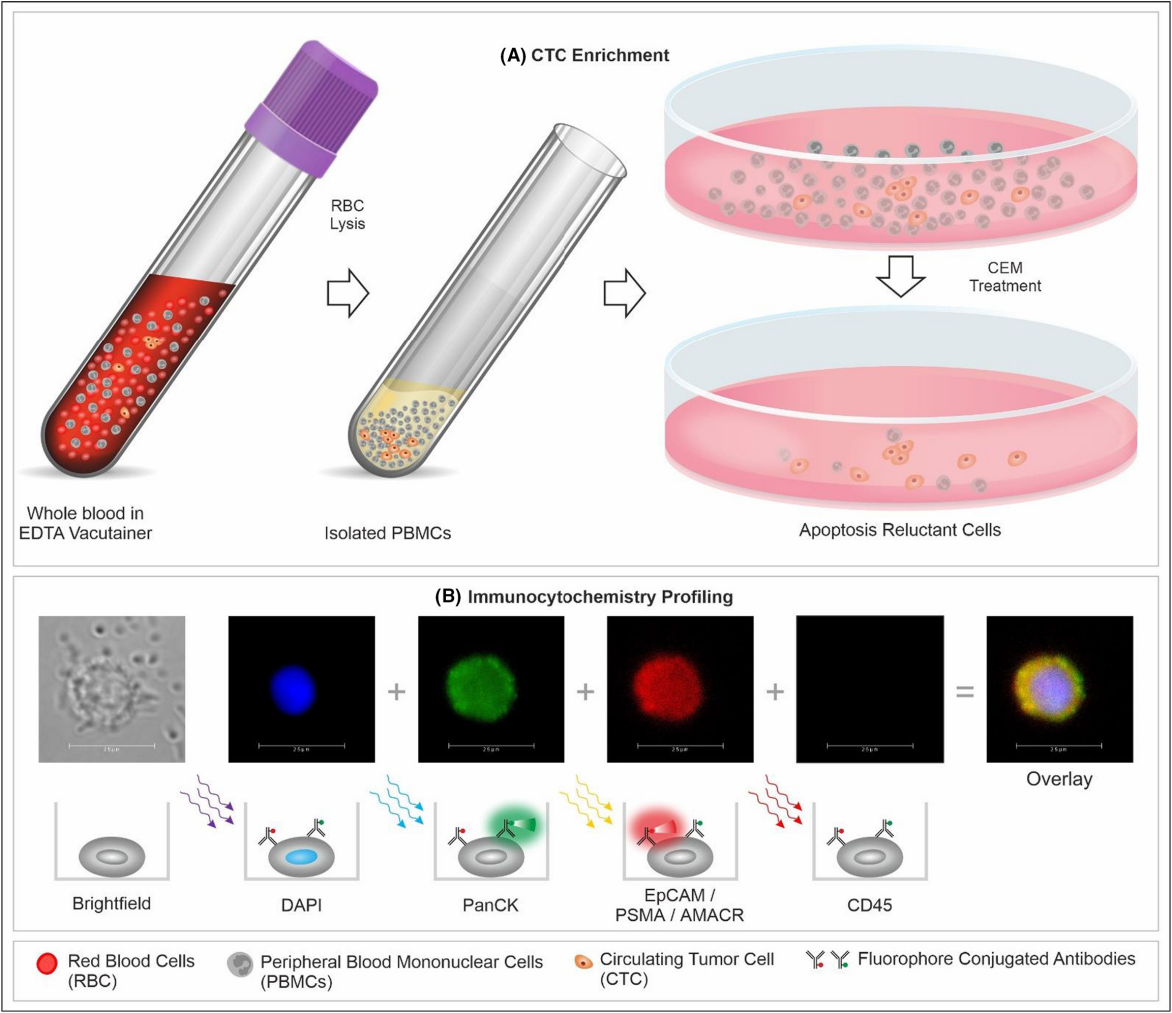
Schema of Test. Functional enrichment of CTCs is achieved using a proprietary CTC enrichment medium (CEM) that eliminates all non‐malignant cells and permits tumor derived malignant cells to survive. Subsequently, the multiplexed immunocytochemistry (ICC) evaluates and identifies PrAD‐CTCs based on positivity of the indicated markers.

**FIGURE 2:**
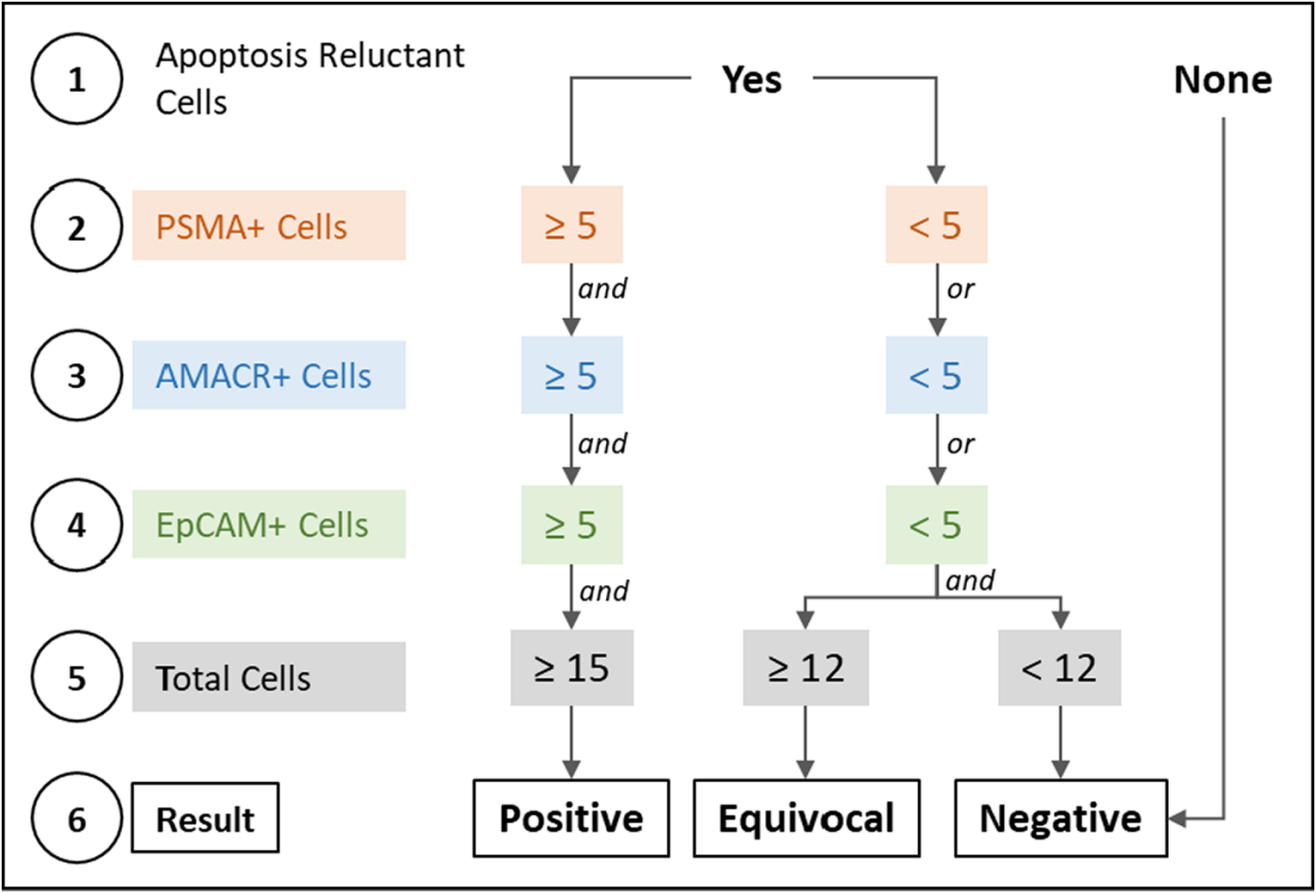
Decision matrix for classifying samples. The detection threshold for PrAD‐CTCs is ≥15 PanCK cells/5 mL, which is constituted by the detection of ≥5 PSMA+ cells, ≥5 AMACR+ cells and ≥5 EpCAM+ cells in the respective aliquots. Priority is given to PSMA and AMACR over EpCAM while classifying samples as “Positive” to ensure specificity for PrAD over other epithelial malignancies where EpCAM+ cells may be detected.

Immunocytochemistry (ICC) profiling of CTC was performed as described previously^26^ and is also provided in Supplementary Materials. A schema showing the various steps of the process including CTC detection and ICC profiling is depicted in Figure 1. The decision matrix for sample classification (“Positive,” “Equivocal,” or “Negative”) based on abundance of each type of marker positive cells is provided in Figure 2. These cut‐offs were based on the Limits of Blank, Detection and Quantitation (LoB, LoD, and LoQ) as determined in the analytical validation studies. The Equivocal classification was assigned to include those samples with up to 20% lower CTC count than the positivity threshold due to losses during storage and transport (as explained in the section on Analyte Stability under analytical validation).

Samples with Equivocal classification were considered positive for the purpose of prostate cancer detection by the test.

### 2.5 Method development and optimization

Comprehensive details of method development and optimization studies as well as their findings are provided in the Supplementary Materials.

### 2.6 Analytical validation

Analytical validations were performed by determining the recovery of reference human prostate cancer cell line (VCaP) spiked into healthy donor blood samples. VCaP reference cells were spiked at various densities as per the design of and requirement for each validation parameter (specified in Supplementary Materials) into healthy donor blood samples, which were processed as per the test for enrichment of CTCs (spiked cells) and immunocytochemistry. The spike‐recovery study design was applicable for validation of analyte stability (and recovery), linearity, limit of detection, limit of quantitation, limit of blank, sensitivity, specificity, accuracy, precision, and interference. Comprehensive details of study design, observations, and inferences are provided in Supplementary Materials.

### 2.7 Case control clinical study

The ability of the Test to detect PrAD cases and differentiate PrAD cases from asymptomatic males was established in a case control study with pre‐biopsy blood samples from 160 recently diagnosed, therapy naïve cases of PrAD and samples from 800 healthy (“asymptomatic”) males aged 49 years and above with neither prior diagnosis nor current suspicion of cancer and with serum PSA ≤0.5 ng/mL. The inclusion and exclusion criteria for this study are provided in Table S8. The asymptomatic cohort was randomized into Training, Test, and Validation Sets in a 60%:20%:20% ratio. The PrAD cases were first segregated by extent of disease as Localized (confined to primary site), Regional (spread to regional lymph nodes), and Distant (metastasized to distal lymph nodes or other organs) for which survival is known.^2^ Subsequently, the stratified cohorts were assigned to Training and Test Sets in a 60%:20%:20% ratio. The Training Set samples comprising of 96 PrAD and 480 healthy males' samples was first evaluated with the analysts unblinded to the status of the samples. Next the blinded Test Set comprising of 32 PrAD and 160 healthy males' samples was evaluated prior to blinded evaluation of the 32 PrAD and 160 healthy males' samples in the Validation Set. Subsequently all Training, Test, and Validation set samples (PrAD and healthy) were shuffled and random 20% samples (extent‐wise for PrAD) were selected for analysis as Validation Set Iteration 2. This shuffling step was repeated to obtain 20 iterations of the Validation Set from which median and range of Sensitivity, Specificity, and Accuracy were reported. The iterative random sampling permitted diverse scenarios with respect to relative proportion of samples with true positive (TP), false negative (FN), true negative (TN), and false positive (FP) findings thus yielding a range of sensitivities and specificities, the median of which was reported. This design eliminates risks of overfitting due to sample enrichment in the Validation Set.

With about 160 cancer samples (cases) and a 90% expected sensitivity (better than 80%), the power of the study for determination of sensitivity is expected to be about 0.95. Similarly, with about 800 asymptomatic samples (controls) in the test set and an expected specificity of 99.9% (better than 99.0%), the power of the study for determination of specificity is expected to be about 0.97. The design of the clinical study is provided in Figure S1.

### 2.8 Prospective clinical study

The performance characteristics of the test were established in a prospective clinical study of blood samples from 210 males with enlarged prostate and urological symptoms who were suspected of PrAD. Additional considerations for deciding the requirement for a prostate biopsy included suspicious findings in digital rectal examination (DRE), ultrasonography (USG), or serum PSA (≥4 ng/mL); in 78 cases, elevated serum PSA was not observed and the indication for a biopsy was based on either DRE or USG in addition to the symptoms. The inclusion and exclusion criteria for this study are provided in Table S10. All participants provided 5 mL blood sample prior to undergoing a prostate biopsy. The findings of the histopathological examination (HPE) and the final diagnosis (cancer or benign) were initially blinded to the sponsor and unmasked only after completion of sample analysis. The concordance between test findings and HPE diagnosis was used to determine Sensitivity, Specificity, and Accuracy. With about 60 cancer cases and an expected sensitivity of 90% (better than 75%), this study design has a power of 0.85. The design of the clinical study is provided in Figure S1.

### 2.9 Molecular concordance studies

In a subset of 20 PrC cases a molecular concordance study was performed on matched tumor tissue and blood samples. Tumor Tissue DNA (ttDNA) was isolated and used for next‐generation sequencing (NGS) profiling using the Ion Proton Platform and the Oncomine Comprehensive Assay v3 Panel to identify gene variants with loss of tumor suppression or gain of oncogenic function which have been previously reported to be significant in/associated with prostate cancer. PBMCs isolated from blood samples were treated with the CEM for CTC enrichment. Genomic DNA (gDNA) was isolated from apoptosis reluctant (surviving) cells and evaluated by a ddPCR assay specific to the detected gene variant on a BioRad QX200 platform. Concordance between tumor tissue and CTCs was determined as the proportion of the latter where the corresponding gene variant was detected by ddPCR.

Tissue samples from the same 20 patients were also evaluated by fluorescence in situ hybridization (FISH) as per manufacturer's protocol for TMPRSS2‐ERG fusion. In samples where tissue was positive for this variation, enriched and harvested CTCs were also evaluated by FISH for the same biomarker.

## 3 RESULTS

### 3.1 Method development

The method development studies showed the viability of multiplexed fluorescence ICC for detection of PrAD‐CTCs with a wide range of EpCAM, PanCK, AMACR, and PSMA expression levels (Figure S2), as well as other key aspects including specificity of marker combination to prostate cancer (Figure S3), absence of PrAD CTCs in benign prostate conditions (Table S1), and the ability of the test to detect CTCs irrespective of patient age (Figure S4), serum PSA levels (Figure S5), Gleason Score (Figure S6), or extent of disease (Figure S7). Comprehensive details are provided in Supplementary Materials.

### 3.2 Analytical validation

Table 1 is a summary of all the findings of the analytical validation study. Analytical validation established analyte stability (Tables S2 and S3), demonstrated high sensitivity, and specificity of the test (Table S4), significant linear characteristics (Figure S8), high precision (Table S5), and no loss of sensitivity in presence of potentially interfering substances (Table S6). Comprehensive details are provided in Supplementary Materials.

**TABLE 1:** Findings of analytical validation studies

	EpCAM, PanCK, CD45	PSMA, PanCK, CD45	AMACR, PanCK, CD45	Overall
Analyte stability	48 h			
Recovery^a^	97.2%	94.4%	94.4%	91.7%
Limit of detection	<1 cell/mL			
Linear range	1–256 cells/mL			
Linearity	*R* ^2^ ≥ 0.99	*R* ^2^ ≥ 0.99	*R* ^2^ ≥ 0.99	*R* ^2^ ≥ 0.99
Sensitivity	95.0%	92.5%	92.5%	92.5%
Specificity	100.0%	100.0%	100.0%	100.0%
Accuracy	97.1%	95.7%	95.7%	95.7%
Precision	CV ≤ 9%	CV ≤ 6%	CV ≤ 6%	CV ≤ 9%
Robustness	CV < 10%			

*Note*: The analytical validation studies established that the Test provides consistent, accurate, and reproducible results with no interference from endogenous or exogenous factors when samples are obtained, stored, and processed under the recommended conditions.

^a^ Above 10 cells/5 mL as determined from the Linearity experiment. Values within parentheses represent 95% CI.

### 3.3 Clinical studies

**FIGURE 3:**
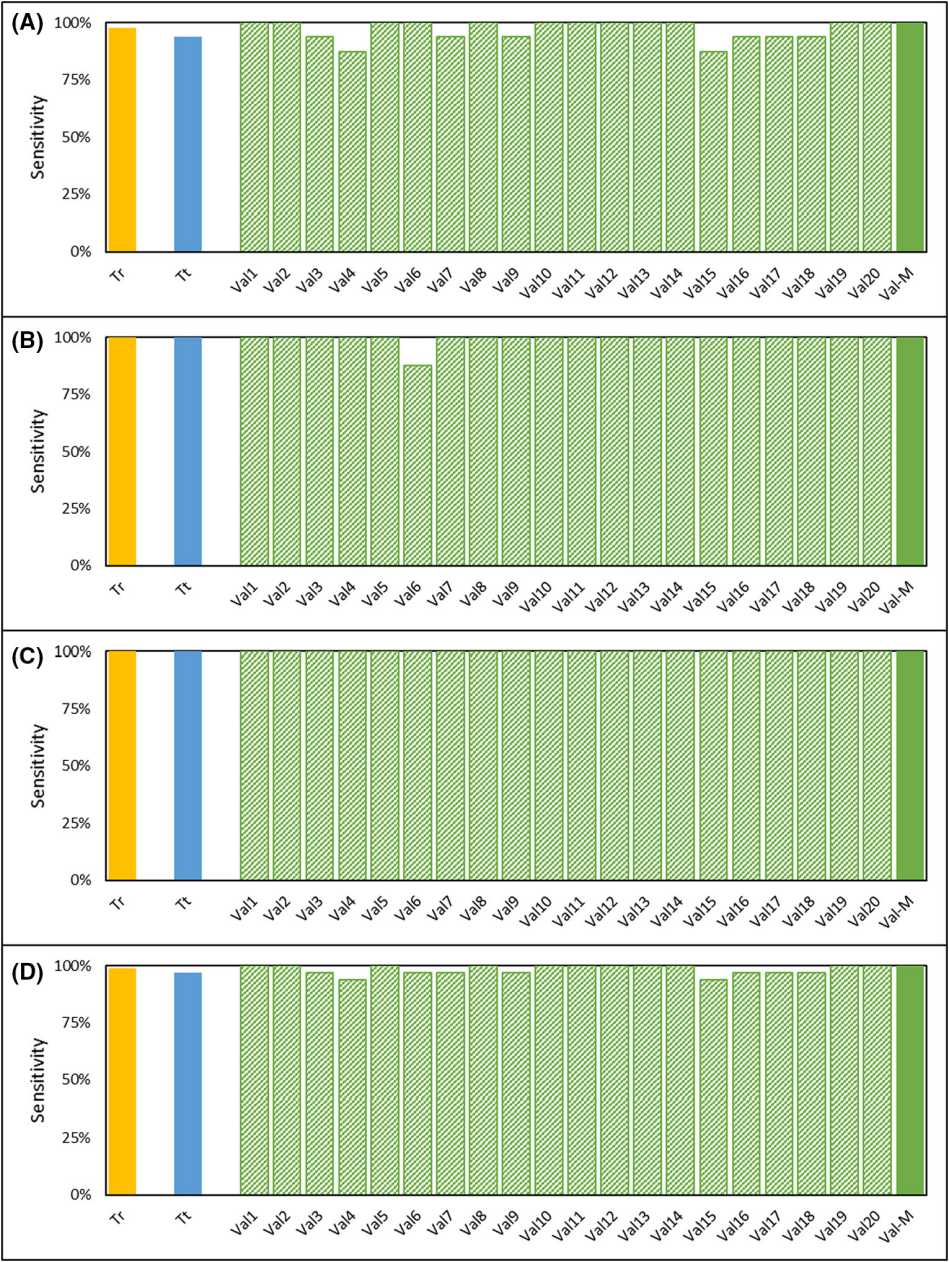
Observed Sensitivity in the Case Control Study. The test initially classifies samples as Positive, Equivocal or Negative based on the Decision Matrix provided in Figure 2. Samples with Equivocal findings are considered as Positive for the purpose of reporting and determination of Sensitivity. Each panel depicts the observed sensitivities in the Training set (Tr, solid orange), Test set (Tt, solid blue), the 20 iterations of the Validation set (Val1‐Val20, green pattern) as well as the median Sensitivity in the Validation set (Val‐M, solid green). The four panels depict findings based on extent of cancer, that is, Local (A), Regional (B), Distal (C), and Overall (D). Table S11 provides a break‐up of the number of Positive, Equivocal, and Negative findings in each of the above sets.

The performance characteristics of the test were established in two clinical studies. The demographics of the study cohorts are provided in Tables S7 and S9 and the inclusion/exclusion criteria are provided in Tables S8 and S10. Both studies were conducted in a South Asian cohort with <0.005% reported prostate cancer incidence,^30^ and also where the prostate cancer risk in asymptomatic males is significantly lower than the <7% reported among Caucasians with ≤0.5 ng/mL serum PSA^31^, ^32^ most of whom are also expected to be clinically insignificant prostate cancer.^31^, ^33^ Due to this low probability of an underlying prostate cancer in healthy subjects, they were a suitable “control” population. Further, the selection of such a control population is also more ethical since it would be unethical to perform a biopsy on asymptomatic individuals for the sole purpose of ruling out prostate cancer for this study. The Case Control Study had a stringent, blinded, iterative cross‐validation design which minimized the risk of overfitting. In this study, the median sensitivity was 100% for local, regional and for metastatic disease as well as overall (Table 2). Figure 3 is a graphical representation of the extent‐wise sensitivities in the Training and Test Sets as well as the 20 iterations of the Validation sets. The break‐up of Positive, Negative, and Equivocal findings in each these sets are provided in Table S11. In absence of any positive or equivocal findings in the asymptomatic cohort, the specificity of the test (cancer v/s healthy) was 100%.

**TABLE 2:** Findings of clinical studies

	Case control study: cancer v/s asymptomatic Specificity: 100.0% (95% CI: *97.7%–100.0%*)		Prospective study: cancer v/s benign Specificity: 100.0% (95% CI: *97.4%–100.0%*)	
	Sensitivity	Accuracy	Sensitivity	Accuracy
Cumulative	100.0% 95% CI: *89.1%–100.0%*	100.0% 95% CI: 98*.1%–100.0%*	91.2% 95% CI: *81.8%–96.7%*	97.14% 95% *CI: 93.9%–98.9%*
Local	100.0% 95% CI: *79.4%–100.0%*	100.0% 95% CI: *97.9%–100.0%*	75.0% 95% CI: 50.9%–91.3%	96.9% 95% CI: 92.9%–98.9%
Regional	100.0% 95% CI: *97.7%–100.0%*	100.0% 95% CI: *97.8%–100.0%*	85.7% 95% CI: 42.1%–99.6%	99.3% 95% CI: 96.3%–99.9%
Distal	100.0% 95% CI: *97.7%–100.0%*	100.0% 95% CI: *97.8%–100.0%*	100.0% 95% CI: 90.8%–100.0%	100.0% 95% CI: 97.9%–100.0%

*Note*: The Stage‐wise and overall performance characteristics of the Test were determined from 20 iterations of the Validation Set in the Case Control Study as well as from the Prospective Study.

In the second (prospective) clinical study with 210 symptomatic males, 68 (32.4%) were eventually diagnosed with PrAD and 142 (67.6%) were diagnosed with benign prostate conditions. There were no positive or equivocal findings among those diagnosed with benign prostate conditions. Hence the specificity of the test (cancer v/s benign) was 100%. Among the 68 cancer cases, the Test assigned 56 samples as positive, six as equivocal and six as negative (Table S12), yielding a sensitivity of 91.2% since equivocals were considered as positive (Table 2). Equivocals were considered as positive for higher PrC detection sensitivity. In the clinical setting, considering equivocals as positive may lead to reduced specificity for PrC (as compared to other cancers where either PSMA or AMACR may be positive), however this improves the chances for detection of PrC or other cancers in such patients who undergo follow‐up investigations. Further, considering equivocals as positive did not decrease the specificity of the test to differentiate PrC cases from asymptomatic individuals. In the prospective study, the sensitivity of the test was observed to correlate positively with Gleason Scores and PSA levels (where available) (Table S13). Among the 68 cancer cases in the prospective cohort were 10 cases with PSA < 10 ng/mL. Of these 10 cases, four were clinically significant with histological grade 3 (*n* = 1, Gleason score 4 + 3) or 4 (*n* = 3, Gleason score 8). The Test was able to detect 75% of these cases.

### 3.4 Molecular concordance studies

Among the 20 tumor samples tested, driver mutations with allele frequency were detected in 15 samples by NGS profiling of tumor tissue DNA using the Oncomine Comprehensive Assay v3 Panel on the Ion Proton Platform. Among these 15 patient samples, a specific TaqMan ddPCR assay was available for variants detected in 12 cases. Genomic DNA was isolated from enriched CTCs and evaluated by ddPCR assays for the corresponding driver mutation (detected on ttDNA by NGS) on a BioRad QX200 platform. Variants in ttDNA detected by NGS were also detected by ddPCR in nine (75%) CTCs (Table S14). A subset of four PrAD cases were identified where the tissue was positive for TMPRSS2‐ERG fusion by FISH. The CTC enriched fraction from these four samples was evaluated by FISH and the TMPRSS2‐ERG fusion was detected in three cases (75%). Overall, the orthogonal concordance studies appeared to confirm that the CTCs detected by the Test originated from the same prostate malignancy. The 75% concordance was considered satisfactory considering clonal diversity in tumor cells and CTCs.

## 4 DISCUSSION

We describe a blood test for Prostate cancer detection based on multiplexed fluorescence ICC profiling of CTCs functionally enriched from a 5 mL blood sample. The test detected Prostate cancer with high sensitivity irrespective of age, serum PSA level, Gleason score, or the extent of disease. Analytical validation ascertained accuracy and reliability of the test. The case control cross‐validation study demonstrated 100% specificity as well as 100% sensitivity across all stages of Prostate cancer. The subsequent prospective clinical validation study demonstrated 91.2% Sensitivity and 100% Specificity in the real world setting for detecting Prostate cancer and differentiating prostate cancer from benign prostate conditions. The Test has high sensitivity for all stages, including early stages as well as high specificity to minimize the risk of false positives. The performance characteristics of the test support its potential clinical utility in Prostate cancer detection.

Serum PSA which is evaluated during standard prostate cancer diagnostic work up in symptomatic men is often assessed as part of elective prostate cancer screening in asymptomatic males.^34^, ^35^ However, PSA testing has lower specificity and is associated with a high false positive rate, for example ~66%.^5^ Other PSA‐based tests such as %‐free PSA,^36^ [−2]pro‐PSA (p2PSA),^37^ and Prostate Health Index (PHI)^38^ with documented sensitivity/specificity trade‐off^36^, ^39^, ^40^ are currently not recommended or approved for routine prostate cancer screening. The inverse relationship between specificity and sensitivity of PSA and PSA‐based tests^40^ implies inefficient triaging where a significant proportion of individuals who do undergo a prostate biopsy based on these tests may actually be free from prostate cancer. Based on the limitations of serum PSA evaluations alone to provide meaningful insight into prostate cancer detection, Thompson et al. suggested that *“PSA levels should no longer be referred to as “normal” or “elevated” but should be incorporated into a multivariable risk assessment to provide individualized risk information for decision making”*.^41^ Among other non‐invasive (blood‐based) approaches, a pan‐cancer detection test based on methylation profiling in cfDNA reported very low sensitivity (~10%) for localized Prostate cancer.^42^, ^43^ While the above tests have been utilized for prostate cancer screening, other tests have been described for triaging of suspected cases so as to improve the specificity of PrC detection and minimize the risk of overdiagnosis. The 4Kscore Test is a follow‐up blood test after an abnormal PSA and/or digital rectal exam (DRE) to determine the probability of aggressive prostate cancer.^44^ The ExoDx™ Prostate Test is a urine‐based test to determine the probability of clinically significant prostate cancer in men with PSA 2–10 ng/mL (“gray zone”) who are considering an initial biopsy.^45^ A recent study by Hugosson et al. demonstrated that the avoidance of systematic biopsy in favor of MRI‐directed targeted biopsy in males with elevated serum PSA levels led to a significant decrease in the risk of overdiagnosis but led to delayed detection of intermediate‐risk PrC in some patients.^46^

Our test is based on detection of CTCs, which are ubiquitous in blood of patients with an underlying solid organ cancer^29^ and unlikely in the blood of individuals without an underlying malignancy as well as those with other non‐malignant or inflammatory conditions. CTCs are hence an ideal analyte to differentiate individuals with and without an underlying malignant condition with high specificity and sensitivity. The risks associated with use of the test are only marginal since it is non‐invasive, requiring only a 5 mL peripheral blood sample. The potential benefits of the test include more effective detection of Prostate cancer and reduced requirement for biopsies in symptomatic males. The strengths of our study include (a) use of adequately powered sample sizes, (b) sample blinding to eliminate bias, (c) an iterative cross‐validation design intended to eliminate risk of over‐fitting, and (d) a prospective study in a real‐world setting. The analytical and clinical validations described in this manuscript provide tangible evidence of the test performance which supports the hypothesis (design) as well as the intended use of the test. The high specificity translates into an exceedingly low risk of false positives in individuals with benign prostate conditions which eliminates or significantly reduces risks of overdiagnosis or overtreatment in these individuals.

Although the test has high performance characteristics for Prostate cancer detection, we note the following potential limitations of the test. Non‐(adeno)‐carcinoma types which account for <8% of Prostate cancer are not detected by this test. The sensitivity for the detection was lower (~75%) for localized Prostate cancer in the prospective study. However, these false negatives would not add to pre‐existing risks since the lower sensitivity for localized cancers can be partially mitigated by the higher sensitivity for subsequent detection at regional stage which has a comparable 5‐year survival.

The risk stratification of prostate cancer includes serum PSA level, clinical stage and Gleason score; a Gleason score of >8 is considered an independent predictor of high‐risk disease with increased rates of treatment failures and poorer outcomes. While test is not intended to provide information on, or correlate with, the Gleason score, it can detect high‐grade/aggressive prostate cancers where early detection is vital for more effective clinical management. As can be seen from the findings in the prospective clinical study, a significant advantage of the test is its ability to detect clinically significant prostate cancers (histological grade 3 or 4) in patients with low serum PSA.

The prospective study had a lower representation of early‐stage disease since it was conducted in a population where prostate cancer is typically detected at advanced stages; of the 68 patients diagnosed with prostate cancer, only 20 (30%) had localized disease (T_1‐3_N_0_M_0_). Since this was an anticipated limitation, the design of the case control study pre‐emptively addressed this challenge by having a higher representation of samples from patients with localized disease; of the 160 patients with prostate cancer, 80 (50%) had localized disease (T_1‐3_N_0_M_0_).

In 78 cases in the prospective cohort, the decision to perform a prostate biopsy despite unremarkable serum PSA (<4 ng/mL) was based on clinical findings/DRE/USG. While this proportion would appear to be higher, they represent standard approaches in India (study location) based on the observations that 15% of symptomatic males with PSA <4 ng/mL are diagnosed with prostate cancer^47^ and that most prostate cancers in India are diagnosed at advanced stages. Notably a prior retrospective cohort analysis reported that 67% of patients were referred for a prostate biopsy at a tertiary centre in Ireland based on abnormal DRE alone.^48^

There would appear to be a minimal risk of overdiagnosis from detection of low‐grade (lower risk) prostate cancers which account for up to 66% of all prostate cancers.^49^ However, since up to 40% of patients initially diagnosed with low‐risk prostate cancer demonstrate pathological progression over time,^50^ detection of low‐grade prostate cancers can benefit from active surveillance.^51^

## 5 CONCLUSION

The high sensitivity and specificity of the test enables prostate cancer detection and differentiation from benign prostate conditions (or healthy individuals) and presents significant advantages over PSA based approaches. The test has potential to reduce the need for invasive biopsies and thus significantly mitigates risks of overdiagnosis and overtreatment. The potential benefits of the test are compelling and support the need for further prospective large cohort clinical studies to determine the performance characteristics of the test for detection of prostate cancer, especially localized disease.

## AUTHOR CONTRIBUTIONS

**Sewanti Limaye:** Conceptualization (equal); methodology (equal); writing – review and editing (equal). **Simon Chowdhury:** Conceptualization (equal); writing – review and editing (equal). **Nitesh Rohatgi:** Conceptualization (equal); writing – review and editing (equal). **Anantabhushan Ranade:** Conceptualization (equal); methodology (equal); writing – review and editing (equal). **Nelofer Syed:** Conceptualization (equal); writing – review and editing (equal). **Johann Riedemann:** Conceptualization (equal); writing – review and editing (equal). **Darshana Patil:** Conceptualization (equal); methodology (equal); project administration (equal); supervision (equal); writing – review and editing (equal). **Dadasaheb Akolkar:** Conceptualization (equal); investigation (equal); methodology (equal); project administration (equal); supervision (equal); writing – review and editing (equal). **Vineet Datta:** Conceptualization (equal); methodology (equal); resources (equal); supervision (equal); writing – review and editing (equal). **Shoeb Patel:** Data curation (equal); formal analysis (equal); investigation (equal); methodology (equal); validation (equal). **Rohit Chougule:** Data curation (equal); formal analysis (equal); investigation (equal); methodology (equal); validation (equal). **Pradyumna Shejwalkar:** Data curation (equal); investigation (equal); validation (equal). **Kiran Bendale:** Conceptualization (equal); investigation (equal); methodology (equal); project administration (equal); validation (equal). **Sachin Apurwa:** Formal analysis (equal); methodology (equal); software (equal); validation (equal); visualization (equal). **Stefan Schuster:** Conceptualization (equal); resources (equal); writing – review and editing (equal). **Jinumary John:** Data curation (equal); formal analysis (equal); validation (equal); visualization (equal); writing – original draft (equal); writing – review and editing (equal). **Ajay Srinivasan:** Data curation (equal); formal analysis (equal); validation (equal); visualization (equal); writing – original draft (equal); writing – review and editing (equal). **Rajan Datar:** Conceptualization (equal); funding acquisition (equal); investigation (equal); methodology (equal); project administration (equal); resources (equal); supervision (equal); visualization (equal); writing – original draft (equal); writing – review and editing (equal).

## FUNDING INFORMATION

No external funding was obtained for this study. The entire study was funded by the Study Sponsor (DCG).

## CONFLICT OF INTEREST STATEMENT

SL, SC, NR, AR, NS, JR have no conflicts of interest to declare. DP, DA, VD, SP, RC, PS, KB, SA, SS, JJ, and AS are in employment of the Study Sponsor (DCG). RD is the founder of the Study Sponsor.

## PRECIS FOR TABLE OF CONTENTS

Enrichment and characterization of circulating tumor cells (CTC) can aid more effective prostate cancer detection. CTC based prostate cancer detection can reduce false negatives and potentially eliminate false positives.

## Supporting information


Data S1

## Data Availability

All relevant data are included in the manuscript and its Supplementary Information file.
